# Minimally Invasive Treatment for Tibial Malrotation after Locked Intramedullary Nailing

**DOI:** 10.1155/2018/4190670

**Published:** 2018-08-23

**Authors:** Kyohei Takase, Sang Yang Lee, Takahiro Waki, Tomoaki Fukui, Keisuke Oe, Tomoyuki Matsumoto, Takehiko Matsushita, Kotaro Nishida, Ryosuke Kuroda, Takahiro Niikura

**Affiliations:** Department of Orthopaedic Surgery, Kobe University Graduate School of Medicine, 7-5-1 Kusunoki-cho, Chuo-ku, Kobe 650-0017, Japan

## Abstract

Rotational malreduction is a potential complication of intramedullary nailing for tibial shaft fractures. We experienced a symptomatic case of a 24° externally rotated malunion that we treated with minimally invasive corrective osteotomy. A 49-year-old man sustained a tibial shaft spiral fracture with a fibula fracture. He had been initially treated elsewhere with a reamed statically locked intramedullary nail. Bone union had been obtained, but he complained of asymmetry of his legs, difficulty walking and running, and the inability to ride a bicycle. We decided to perform corrective osteotomy in a minimally invasive fashion. After a 1 cm incision was made at the original fracture site, osteotomy for the affected tibia was performed with an osteotome after multiple efforts at drilling around the nail with the aim of retaining it. Fibula osteotomy was also performed at the same level. Two Kirschner wires that created an affected rotational angle between the fragments were inserted as a guide for correction. The distal locking screws were removed. Correct rotation was regained by matching the two wires in a straight line. Finally, the distal locking screws were inserted into new holes. The patient obtained bony union and has returned to his preinjury activities with no symptoms.

## 1. Introduction

Intramedullary nailing, the most common method for repairing tibial shaft fractures [1], has the advantage of requiring only minimal surgical dissection with appropriate preservation of blood supply to the fracture. The surgical implant offers biomechanical fracture stabilization and acts as a load-sharing device, allowing early postoperative mobilization [2]. Some reports, however, have described complications associated with the procedure [3, 4]. A potentially serious, often underappreciated, complication of this procedure is rotational malreduction. In addition to presenting cosmetic dissatisfaction, torsional deformities may lead to a variety of disorders [3, 4]. For example, it has been reported that greater degrees of tibial malunion can lead to degenerative arthritis of the adjacent joint [5]. When a symptomatic rotational deformity occurs after repair using an intramedullary nail, corrective osteotomy should be done. Although several corrective surgical techniques have been reported [6–8], most require longer operation times and are more invasive. We describe a case of tibial fracture malunion treated by osteotomy while retaining the nail in a minimally invasive fashion.

## 2. Case Report

**Figure 1:**
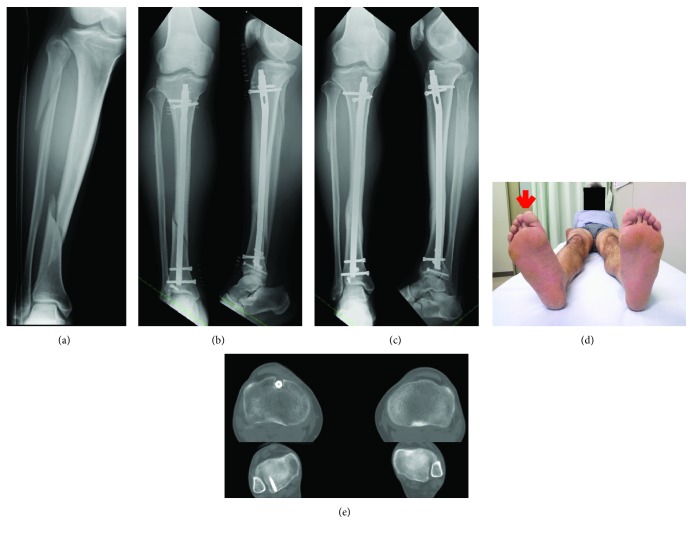
(a) Initial posttraumatic anteroposterior radiographs of the right tibia and fibula. (b-c) Anteroposterior and lateral radiographs of the right tibia obtained (b) immediate postoperatively and (c) 1 year postoperatively. (d) Photograph. (e) Computed tomography (CT) images of the right and left tibia of the patient show external tibial torsion.

A 49-year-old man sustained a tibial shaft spiral fracture (AO/OTA classification 42-A2) with a fibular fracture (Figure 1(a)). He had no previous medical history. The fracture was treated initially at another hospital with a reamed statically locked intramedullary nail (Figure 1(b)). He noted increased external rotation of the affected leg immediately after the surgery. The tibial fracture united after a year (Figure 1(c)), but he still complained of the asymmetry of his legs, difficulty walking and running, and inability to ride a bicycle. Computed tomography (CT) of both tibias showed 24° of increased external rotation of the affected leg (Figures 1(d), 1(e)). Because it was a symptomatic rotational deformity, we decided to perform corrective osteotomy in a minimally invasive fashion.

**Figure 2:**
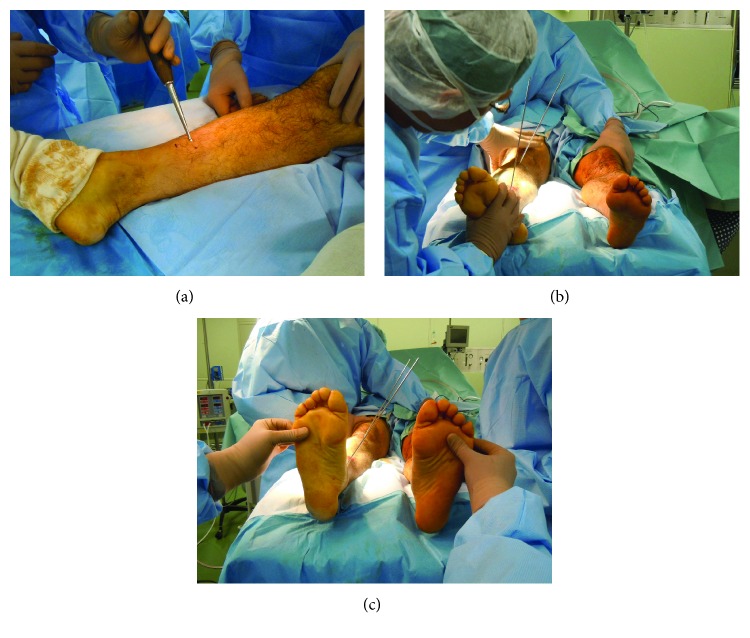
Photographs. (a) Small incision at the original fracture site. (b) Two 3.0 mm Kirschner wires were inserted as guides for correction. (c) Kirschner wires used as a guide for correcting the angle between the proximal and distal bones, which are matched in a straight line.

**Figure 3:**
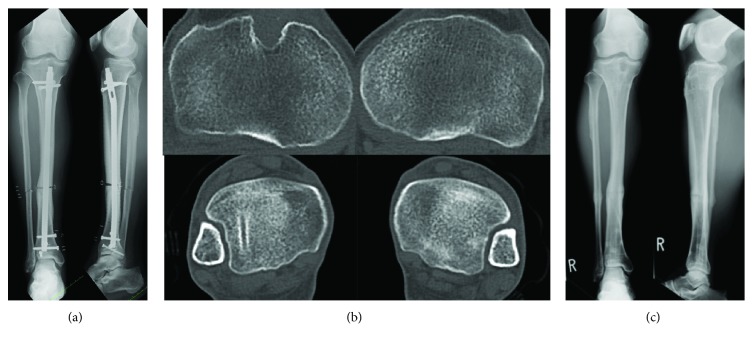
(a) Anteroposterior and lateral radiographs of the right tibia obtained immediately after corrective osteotomy. (b) CT images of the right and left tibias obtained 1 year after corrective osteotomy. (c) Anteroposterior and lateral radiographs of the right tibia obtained 5 years after corrective osteotomy.

The surgical procedure consisted of, first, a 1 cm skin incision at the original fracture site. Multiple efforts were then made to drill around the nail in a radial manner (leaving the nail in place) while using a 3.0 mm Kirschner wire to prepare a percutaneous osteotomy line. Osteotomy for the affected tibia was performed percutaneously using an osteotome on the prepared osteotomy line while retaining the intramedullary nail (Figure 2(a)). Fibular osteotomy was also done at the same level. Next, two 3.0 mm Kirschner wires, which created a 24° rotational angle in the axial plane between the bone fragments, were inserted as guides for correction (Figure 2(b)). The distal locking screws were then removed. After matching the two Kirschner wires in a straight line, correct rotation was confirmed (Figure 2(c)). We assessed the rotational correction intraoperatively to evaluate both sides of the thigh-foot angle [9, 10]. Finally, the distal three locking screws were inserted into holes different from the original hole (Figure 3(a)). At 1 year postoperatively, the patient obtained bony union and has returned to his preinjury activities with no symptoms. The implant was removed 1 year postoperatively on the patient's demand. The appropriate correction of the rotational deformity was confirmed on a CT scan (Figure 3(b)). Postoperative follow-up was continued until 5 years after the corrective osteotomy (Figure 3(c)). The patient was still free from any symptoms and had full range of hip, knee, and ankle motion.

## 3. Discussion

Tibial malrotation after intramedullary nailing is likely more common than is reported [6]. A clinical evaluation showed that it occurred with a frequency of about 0–7% [11, 12]. A CT study reported rotational malreduction of >10° in 20–25% of patients after nailing [3]. Some studies have identified a significant malrotation difference of >10° compared with the unaffected leg [3, 13]. Malrotation of the tibia can lead to significant limitation of function, secondary to hindfoot disability and the development of early degenerative arthritis [7, 14]. Although other previous reports have reported indications for corrective osteotomy, its use is still controversial [6, 7, 15]. For example, the range of acceptable malrotation is reportedly <15° [6]. In contrast, another report stated that a rotational deformity of <20° did not usually produce any handicap [15]. Because our patient complained of both cosmetic (asymmetry of his legs) and functional (difficulty walking and running, inability to ride a bicycle) problems, we performed corrective surgery. Several corrective surgical techniques have been reported [6, 8, 16]. For example, after removing the intramedullary nail, a proximal (or distal) derotational osteotomy was performed and a static locked intramedullary nail placed [6]. In another, after removing the intramedullary nail, a supramalleolar derotational osteotomy was performed and plate fixation accomplished [16]. A third possibility is after removing the intramedullary nail, an osteotomy is performed and an Ilizarov external fixator is applied for lengthening and rotational correction [8]. For each of these techniques, however, there is a drawback: the intramedullary nail must be removed. In addition, attaching an Ilizarov external fixator takes longer to perform.

Strecker et al. have reported a technique wherein the originally implanted nail can be preserved in corrective osteotomy for rotational femoral malunion [17]. We applied this technique to tibial malrotation after intramedullary nailing because minimally invasive surgery is always the preferable option. Consequently, we obtained a good clinical result. As the surgical method described in our case can be carried out in a minimally invasive fashion, we believe it should be considered an effective option for treating tibial malrotation after nailing.
